# Loss of PIG3 increases HIF-1α level by promoting protein synthesis *via* mTOR pathway in renal cell carcinoma cells

**DOI:** 10.18632/oncotarget.8401

**Published:** 2016-03-27

**Authors:** Guang Chen, Jin-Ye Xu, Jie Chen, Jian-Xin Zhang, Jun Zhou, Yong Liang, Xiao-Fei Ding

**Affiliations:** ^1^ School of Pharmaceutical and Chemical Engineering, Taizhou University, Taizhou, Zhejiang, China; ^2^ Institute of Tumor, Taizhou University, Taizhou, Zhejiang, China; ^3^ Laboratory of Cancer Biology, Provincial Key Lab of Biotherapy in Zhejiang, Sir Runrun Shaw Hospital, Medical School of Zhejiang University, Hangzhou, China; ^4^ Taizhou Municipal Hospital, Taizhou, Zhejiang, China; ^5^ School of Medicine, Taizhou University, Taizhou, Zhejiang, China

**Keywords:** PIG3, HIF-1α, mTOR, renal cell carcinoma, migration

## Abstract

PIG3 is a target of the tumor suppressor p53 and is thought to be involved in p53-mediated cell apoptosis. Although PIG3 is similar to oxidoreductases involved in generating ROS, whether PIG3 would regulate HIF-1α was never characterized directly. Here we demonstrated that knockdown of PIG3 by transfecting with specific siRNA could increase the expression of HIF-1α in several human cancer cell lines, including CAKI, FTC-133 and A549. It indicates that PIG3 may be involved in the regulation of HIF-1α. Furthermore, we revealed that PIG3-siliencing increased HIF-1α protein level through promoting its protein biosynthesis via mTOR pathway. In addition, the effect of PIG3 on the production of HIF-1α was further related to VEGF secretion and cell migration. PIG3-downregulation increased the secretion of VEGF and promoted the migration of renal cancer cells obviously. Taken together, these data suggest that PIG3 was involved in HIF-1α regulation, and reveal a novel signaling pathway of PIG3/HIF-1α in the regulation of cell migration in renal cell carcinoma.

## INTRODUCTION

Hypoxia-inducible factor-1α (HIF-1α) is a major transcription factor responsible for the induction of hypoxia-response elements (HREs)-containing genes that facilitate adaptation and survival of cells and the whole organism under hypoxia [1]. To date, there are more than 100 hypoxia-inducible genes identified with varying functions, including those involved in erythropoiesis/iron metabolism, angiogenesis, glucose, metabolism, cell proliferation/survival and apoptosis [2, 3]. Overexpression of HIF-1α was found in various human cancers, HIF-1α downstream genes have been identified that are widely involved in the malignant features of tumors, including angiogenesis, invasion, metastasis, and drug resistance [4–6].

HIF-1α is regulated in both oxygen-dependent and oxygen-independent manner. The ubiquitination-mediated degradation is the most important regulator of HIF-1α levels. In normoxia, hydroxylation of two proline residues and acetylation of a lysine residue in its oxygen-dependent degradation domain (ODDD) promote the association of HIF-1α with the von Hippel-Lindau (pVHL) ubiquitin E3 ligase complex, leading to HIF-1α degradation *via* ubiquitin-proteasome pathway [7–9]. Besides pVHL, p53, HSP90, cJun, etcetera, have also been found to relate to HIF-1a ubiquitination and stability [10]. In addition, the mitogen-activated protein kinase (MAPK) pathway and PI3K/AKT/mTOR pathway seem to play important role in HIF-1α expression [11–13]. Here, we found that knockdown of PIG3 mediated by siRNA transfection increased HIF-1α protein level.

PIG3 (p53 inducible gene 3), also called TP53I3 (tumor protein p53-inducible protein 3), is one of the P53 protein target originally indentified by Polyak et al [14]. To date, PIG3 has been found to participate in apoptosis, the generation of ROS, DNA damage response and mediating cancer cell death [15–17]. It is well known that PIG3 is a target of p53, but the down-stream signaling pathway of PIG3 is poorly understood.

In the present study, we showed that PIG3 may be involved in HIF-1α regulation. And PIG3 knockdown mediated by RNAi could up-regulate VEGF secretion *via* HIF-1α to promote renal cancer cell migration, which contributes to improve our knowledge of PIG3 function and HIF-1α regulation.

## RESULTS

### PIG3 regulates cellular HIF-1α protein level

**Figure 1:**
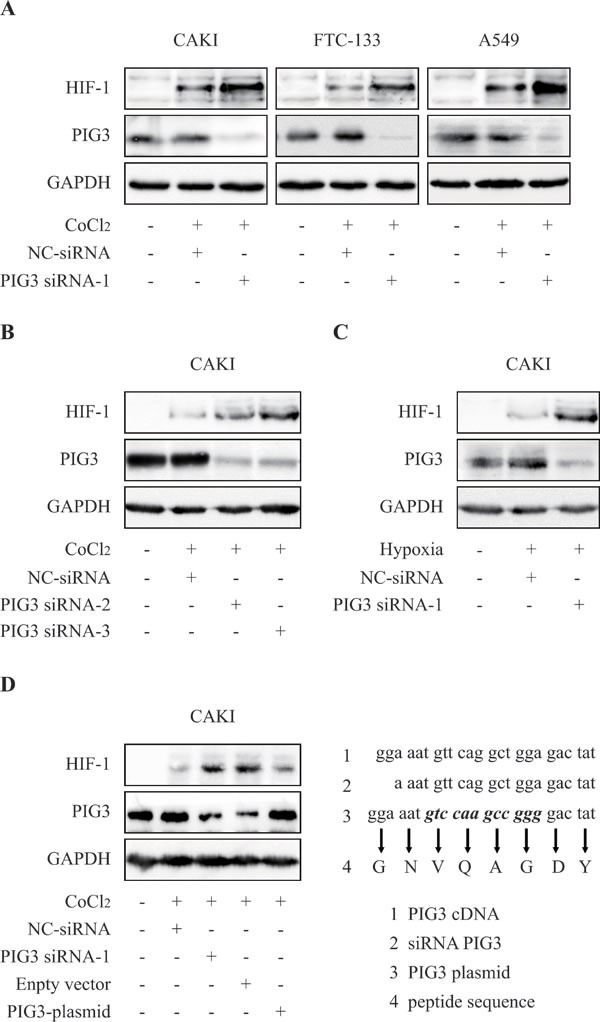
Knockdown of PIG3 up-regulates HIF-1α **A.** PIG3 down-regulation mediated by siRNA-transfection significantly increased HIF-1α protein level in CAKI, FTC-133 and A549 cells. Cells were transfected with PIG3-siRNA or negative control siRNA for 48 h before CoCl_2_-induced hypoxia treatment. **B.**, treatments with both PIG3 siRNA-2 and PIG3 siRNA-3 led to HIF-1α increase in CAKI cells under CoCl_2_-induced hypoxia-mimic system for 6 hours. **C.**, HIF-1α in PIG3 siRNA-1-transfected CAKI cells also increased under hypoxia (1% O_2_) for 6 h. **D.** Complementation with PIG3 blocked HIF-1α increase induced by PIG3-siRNA. CAKI cells were transfected with the mutant PIG3 plasmid 24 h after PIG3-siRNA treatment. Protein levels were analyzed by Immunoblotting, GAPDH was employed a loading control. All the experiments above were conducted thrice.

We first investigated whether PIG3 regulated HIF-1α expression in human renal cell carcinoma cell. Knockdown the PIG3 expression by transfecting *pig3*-specific siRNA in CAK-I cells, then the PIG3-silencing cells were exposed to CoCl_2_-induced hypoxia-mimic system for 6 hours to detect the HIF-1α protein. As shown in Figure 1A, the protein levels of cellular HIF-1α were obviously higher than those in the hypoxic negative control cells. Similar results were found in other cell lines, such as follicular thyroid cancer FTC-133 cells and non-small-cell lung cancer A-549 cells.

Moreover, the similar profiles were showed by transfecting two different siRNAs targeting PIG3 into CAKI cell (Figure 1B) and the same story was found in hypoxic condition under 1% O_2_ (Figure 1C).

Furthermore, re-expression of PIG3 in siRNA experiment was done to rescue the phenotype of HIF-1α increase, avoiding off target-effects. We conducted PIG3 complementation experiments by transfecting a plasmid carrying a point mutant PIG3 sequence (Figure 1D) into the PIG3-silenced CAKI cells. This plasmid expresses same protein, but its transcripts could not be disrupted by PIG3-siRNA due to their unpairing (Figure 1D). PIG3 complementation effectively blocked the increase of HIF-1α protein levels induced by PIG3 loss (Figure 1D), indicating that PIG3 plays a causal role in the control of cellular HIF-1a protein levels.

### Knockdown of PIG3 Up-regulates HIF-1α level through promoting protein biosynthesis

**Figure 2:**
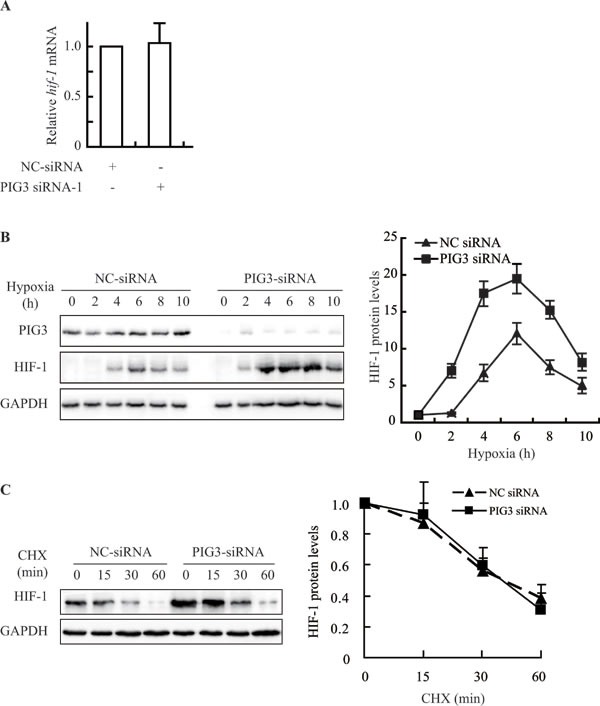
Knockdown of PIG3 up-regulates prevents HIF-1α by promoting protein biosynthesis **A.**, Real-Time PCR analysis showed that PIG3-silencing in CAKI cells did not increase HIF-1α mRNA level. Data shown are fold-change relative to the HIF-1α levels in cells transfected with negative control siRNA (with normalization relative to GAPDH levels). **B.**, Silencing PIG3 in CAKI cells affected the kinetics of the HIF-1α protein when the cells were exposed to hypoxic conditions. **C.**, PIG3 silencing did not affect the degradation of HIF-1α protein under hypoxia. Forty-eight hours after transfection with PIG3-siRNA or negative control siRNA, CAKI cells were pretreated under hypoxia for 4 h followed by treatment with 100 μg/mL cycloheximide (CHX) to block protein synthesis for the indicated times. The mean values from two experiments are connected by the lines. Protein levels were analyzed by Immunoblotting, GAPDH was employed a loading control. All the experiments above were conducted at least twice.

HIF-1α is regulated by various pathways at different levels [18]. Firstly, we detected the levels of HIF-1α mRNA in the PIG3-knockdown CAKI cells to examine whether PIG3 affects the transcription of HIF-1α. However, PIG3 silencing did not change the level of HIF-1α mRNA, indicating that PIG3 does not affect the transcription of the HIF-1α gene (Figure 2A).

Secondly, we investigated the alteration kinetics of the HIF-1α protein levels in CAKI cells transfected with negative control or PIG3 siRNA responding to hypoxia. As shown in Figure 2B, in PIG3-knockdown CAKI cells, the levels of HIF-1α protein went up earlier than those in the control cells on exposure to hypoxia. HIF-1α protein levels increased significantly at the 2-hour time point in PIG3-silencing cells. In contrast, in the control cells, at the same 2-hour time point, the levels of HIF-1α protein was much lower (Figure 2B). The result suggests that the loss of PIG3 is likely to promote the production of the HIF-1α protein.

To further examine whether the induction of HIF-1α protein in PIG3-knockdown cells is associated with its degradation, we exposed the cells to the protein biosynthesis inhibitor cycloheximide (CHX). The HIF-1α protein levels changed in the similar pattern in both cell (Figure 2C). The data indicate that PIG3 loss did not inhibit the protein degradation of HIF-1α.

### PIG3-siliencing promotes the production of HIF-1α *via* PI3K/mTOR pathway

**Figure 3:**
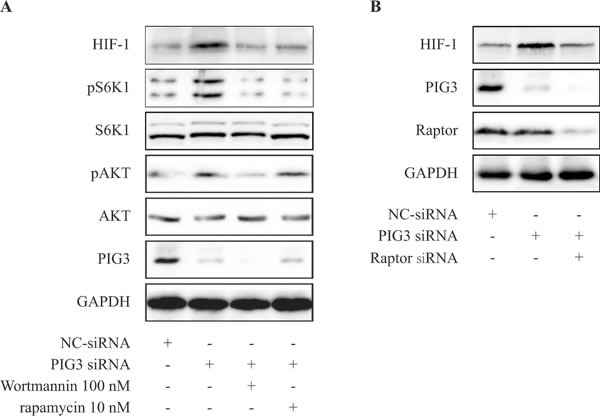
PIG3-silencing promotes the production of HIF-1α *via* PI3K/mTOR pathway **A.** CAKI cells were transfected with PIG3-siRNA for 24 h, then treatment with rapamycin 10 nM for 24 h or wortmannin 100 nM for 8 h before hypoxia treatment. **B.** CAKI cells were transfected with PIG3-siRNA for 24 h, then treatment with Raptor siRNA for 24 h before hypoxia treatment. All the experiments above were conducted thrice.

It well known that PI3K/Akt/mTOR signaling pathway mediates HIF-1α translation in various cancer cells [11, 12, 19, 20]. We examined whether compounds targeting PI3K/mTOR pathway would attenuate the production of HIF-1α protein induced by PIG3-knockdown. As shown in Figure 3A, treatment of rapamycin at 10 nM for 24 h or wortmannin 100 nM for 8 h significantly inhibited PIG3-silencing-stimulated HIF-1α going up in CAKI cells.

To further determine the role of mTOR in PIG3-loss-induced HIF-1α expression, we examined up-regulation of HIF-1α protein level induced by PIG3 loss after down-regulation of Raptor by transiently transfection of siRNA. As shown in the Figure 3B, down-regulation of Raptor significantly inhibited PIG3-loss-induced increase of HIF-1α protein level. These results indicated that down-regulation of PIG3 may induce HIF-1α translation in hypoxia *via* mTOR pathway.

### PIG3-silencing promotes the VEGF secretion and migration of renal cancer cells

**Figure 4:**
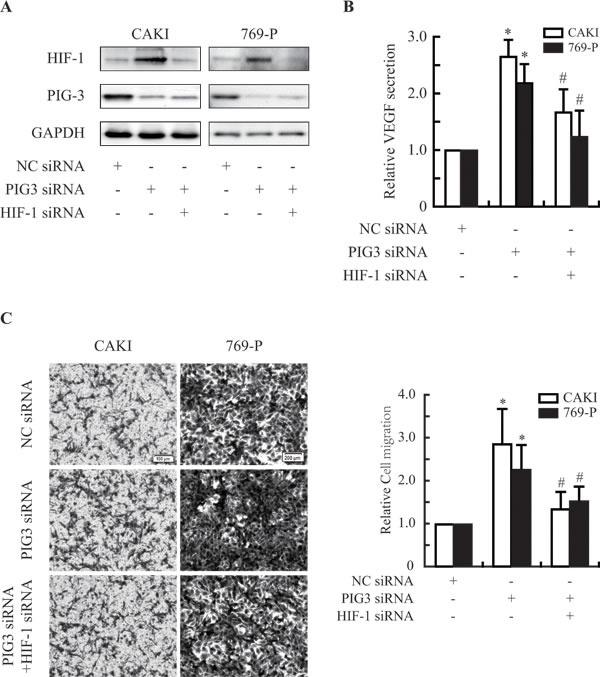
PIG3-silencing promotes the secretion of VEGF and the migration of CAKI and 769-P cells **A.**, Knocking down PIG3 and/or HIF-1α by transfecting with siRNA. **B.**, ELISA assays showed that down-regulation of PIG3 led to an increase in the secretion of VEGF *via* HIF-1α in CAKI and 769-P cells exposed to hypoxia for 12 h. **C.**, PIG3-silencing promoted CAKI and 769-P cells migration by targeting HIF-1α. All the experiments above were conducted thrice. Columns indicate the mean of three experiments; Bars, S.D.

Vascular endothelial cell growth factor (VEGF) is one of the major target genes HIF-1α [6, 21]. To test whether PIG3 knockdown impairs HIF-1α biological function, we detected the VEGF secretion in the PIG3-silenced CAKI and 769-P cells. As shown in Figure 4A, Silencing PIG3 significantly increased the protein secretion of VEGF under hypoxic conditions. VEGF is the most potent endothelial-specific mitogen and is known to directly participate in angiogenesis and metastasis [20, 22–25]. To validate the role of PIG3 in metastasis-related events, we detected the migration of PIG3-silenced CAKI and 769-P cells. Knocking down PIG3 obviously increased the migration activities of CAKI and 769-P cells. However, HIF-1α-knockdown obviously blocked the increase of cell migration induced by PIG3 loss (Figure 4C), indicating that PIG3-loss increases cell migration in HIF-1a dependent way to some extent.

## DISCUSSION

In the present study, we showed that down-regulation of PIG3 by specific siRNA transfection induced the expression of HIF-1α in CAKI cells. Similar results were replicated in another human cancer FTC-133 and A549 cell lines. PIG3 appeared to involve in regulating HIF-1α. Moreover, we found that knockdown of PIG3 would increase HIF-1α protein Level *via* promoting its protein biosynthesis mediated by mTOR pathway. Furthermore, PIG3-silencing increased the secretion of VEGF and promoted the migration of renal cancer cells.

The relationship between p53 and HIF-1α has been the subject of several studies, which significantly affect cancer progression and compromise treatment outcomes [24]. Kaluzova et al. reported that activated p53 mediates an accelerated degradation of HIF-1α protein, without affecting significantly HIF-1α transcription [26]. While Munekazu et al. focused on P53-induced microRNA-107 inhibiting HIF-1 transcription and tumor angiogenesis [27]. It is also reported that p300 is related to the crosstalk between HIF-1 and p53 on the level of trans-activation [28]. It seemed that P53 regulates HIF-1α at various levels *via* different pathways [26–34]. As a down-stream target of p53, the PIG3 is mostly used as a long lived proapoptotic marker [14], and has also been shown to participate in the DNA damage response recently [15]. Although PIG3 activation leads to the ROS generation [16], there is no direct evidences show that PIG3 would regulate HIF-1α. Here we present the evidence to demonstrate that PIG3 functions as a new regulator of HIF-1α. Furthermore, we detailed that loss of PIG3 led to accumulation of HIF-1α by promoting the HIF-1α protein biosynthesis *via* PI3K/mTOR pathway.

VEGF is a main target of HIF-1α and has various effects, including inducing angiogenesis and promoting cell migration [21, 35]. In this regard we have demonstrated that knockdown of PIG3 promoted VEGF secretion and migration activity of renal cell carcinoma CAKI and 769-P cells.

In conclusion, our data provide more insights into of PIG3`s role in HIF-1α regulation, and the regulation network of the cellular HIF-1α. This will help us to understand the tumor progression as well as develop new therapeutic approaches.

## MATERIALS AND METHODS

### Cell culture and reagents

CAKI, FTC133 and A549 cells were obtained from ATCC and maintained in appropriate medium as suggested by ATCC. Cells were incubated in a humidified atmosphere of 95% air plus 5% CO_2_ at 37 ℃. CoCl_2_ was obtained from Zhiyuan Chemical Reagent Co., Ltd. (Tianjin, China). MG-132 and CHX were obtained from MCE (Shanghai, China). Rapamycin was obtained from LC Laboratories (Woburn, MA, USA). Wortmannin was purchased from Selleckchem.cn (Shanghai, China)

### Transfection of siRNA

Synthetic siRNA were purchased from Shanghai GenePharma Co., Ltd. The siRNA was transfected into cells using siRNA-Mate (Shanghai GenePharma Co., Ltd, Shanghai, China) according to the instructions of the manufacturer, with sequences as follows: *pig3* : sense 5-AAAUGUUCAGGCUGGAGACUAdTdT-3. 5-UAGUCUCCAGCCUGAACAUUUdTdT-3; hif-1α:5-TACGTTGTGAGTGGTATTATT; 5-CUGAUGACCAGCAACUUGATT.

### Immunoblotting

Immunoblotting was conducted with standard procedures [36], using antibodies against PIG3 (Origene, Rockville, MD), HIF-1α and GAPDH (Santa Cruz Biotechnology, Santa Cruz, CA), p-AKT, AKT, p-S6K1, S6K1 (Cell Signaling Technology, Beverly, MA).

### Real-time quantitative PCR

Total RNA was extracted with Trizol according to the manufacturer's instructions and was transcribed using Prime ScriptTM RT reagent Kit (TaKaRa, Dalian, China). The cDNA template was amplified by real-time PCR using SYBR-PremixExTaqTM Kit (TaKaRa, Dalian, China). The primer sequences were as follows: 5′- TCATCCAAGAAGCCCTAACGTG -3′(forward), 5′- TTTCGCTTTCTCTGAGCATTCTG -3′(reverse) for *hif-1α*, 5′-GCACCGTCAAGGCTGAGAAC-3′ (forward), 5′-GCCTTCTCCATGGTGGTGAA-3′ (reverse) for GAPDH. Thermal cycling was programmed as follows: 95℃ for 30 sec followed by 40 cycles of 95℃ for 5 sec, 60℃ for 20 sec and 72℃ for 15 sec, and then 72℃ for 10 min. Gene expression was assessed by delta Ct method and mRNA levels of HIF-1α were normalized to those of GAPDH internal standard.

### Transwell assay

Cell migration was evaluated using an 8-mm pore size Transwell system (Costar, Cambridge, MA, USA). Briefly, Cells were resuspended in serum-free RPIM-1640 at a density of 2×10^5^ cells/mL. The top chamber of transwell was loaded with 100 μL of cell suspension and the bottom chamber was loaded with 0.6 mL of RPIM-1640 containing 10 % FBS. The total migrated cells to the lower chamber were fixed, stained with 0.1% crystal violet, and photographed after treatment. Crystal violet stained cells were dissolved with 10% acetic acid and OD value was measured at 595 nm.
